# The *Plasmodium falciparum* CCCH Zinc Finger Protein ZNF4 Plays an Important Role in Gametocyte Exflagellation through the Regulation of Male Enriched Transcripts

**DOI:** 10.3390/cells11101666

**Published:** 2022-05-17

**Authors:** Borja Hanhsen, Afia Farrukh, Gabriele Pradel, Che Julius Ngwa

**Affiliations:** Division of Cellular and Applied Infection Biology, Institute of Zoology, RWTH Aachen University, 52074 Aachen, Germany; borja121985@googlemail.com (B.H.); afia.farrukh@bio2.rwth-aachen.de (A.F.); pradel@bio2.rwth-aachen.de (G.P.)

**Keywords:** *Plasmodium falciparum*, CCCH-ZFP, RNA metabolism, ZNF4, exflagellation, malaria transmission

## Abstract

CCCH zinc finger proteins (ZFPs) function mainly as RNA-binding proteins (RBPs) and play a central role in the mRNA metabolism. Over twenty seven CCCH-ZFPs are encoded in the genome of the human malaria parasite *Plasmodium falciparum*, the causative agent of malaria tropica. However, little is known about their functions. In this study, we characterize one member of the PfCCCH-ZFP named ZNF4. We show that ZNF4 is highly expressed in mature gametocytes, where it predominantly localizes to the cytoplasm. Targeted gene disruption of *ZNF4* showed no significant effect in asexual blood stage replication and gametocyte development while male gametocyte exflagellation was significantly impaired, leading to reduced malaria transmission in the mosquito. Comparative transcriptomics between wildtype (WT) and the ZNF4-deficient line (ZNF4-KO) demonstrated the deregulation of about 473 genes (274 upregulated and 199 downregulated) in mature gametocytes. Most of the downregulated genes show peak expression in mature gametocyte with male enriched genes associated to the axonemal dynein complex formation, and cell projection organization is highly affected, pointing to the phenotype in male gametocyte exflagellation. Upregulated genes are associated to ATP synthesis. Our combined data therefore indicate that ZNF4 is a CCCH zinc finger protein which plays an important role in male gametocyte exflagellation through the regulation of male gametocyte-enriched genes.

## 1. Introduction

Malaria is one of the deadliest parasitic diseases and has resulted in over 241 million infections and 627,000 deaths in 2020 [1]. The disease is caused by apicomplexan parasites of the genus *Plasmodium*, with *P. falciparum* being the causative agent of malaria tropica, causing the most severe form. Malaria is transmitted from the human to the anopheline mosquito by a subset of specialized cells, the gametocytes, which leave the asexual replication cycle and differentiate into male and female forms. Once mature, the gametocytes are picked up by a blood-feeding mosquito. In the midgut of the mosquito, they become activated by external stimuli, then the male gametocytes undergo exflagellation, a process that includes three rounds of DNA replication followed by the release of eight motile microgametes, while the females develop into macrogametes. Following the fusion of a microgamete and a macrogamete, a zygote forms during the first hour post-activation, which transforms into an infective ookinete within the following 24 h [2]. The motile ookinete traverses the midgut epithelium before settling down and forming an oocyst between epithelium and basal lamina [2,3].

Gametocyte development as well as activation in the mosquito midgut is supported by well-coordinated sequences of gene activation and silencing events, which are essential to prepare the parasite for transmission from the human to the insect host. Research over the past decade has demonstrated a pivotal role of transcriptional and translational regulation in this process. Gametocyte commitment, the process by which asexual blood stage parasites enter the sexual pathway to form gametocytes, has been shown to be regulated by the transcription factor AP2-G. The expression of AP2-G promotes the transcription of early gametocyte genes, which leads to gametocyte commitment and formation [4,5,6]. In a recent study, it was demonstrated that another transcription factor AP2-G5 is essential for gametocyte maturation through the downregulation of AP2-G and a set of genes activated by AP2-G prior to gametocyte development [7]. Other transcription factors such as AP2-FG and AP2-O3 have been shown to regulate gene expression in female gametocytes [8,9], and the AP2-O family regulates ookinete development [10,11].

Translational repression is an important mechanism of transcript regulation and has been shown to play an important role in the regulation of the female gametocyte transcripts of the malaria parasite. Transcripts of the parasite required for mosquito midgut stage formation are synthesized and stored in granules in female gametocytes, where they are translationally repressed by binding to regulatory ribonucleoprotein complexes, and the repression is only released after gametocyte activation to promote zygote to ookinete formation [12,13]. In *P. berghei*, the RNA helicase development of zygote inhibited (DOZI) and the Sm-like factor CITH (homolog of worm CAR-I and fly Trailer Hitch) play central roles in the formation of the ribonucleoprotein complex, which stores several transcripts including P25 and P28, which are later released after gametocyte activation for zygote to ookinete development [14,15]. In *P. falciparum*, on the other hand, the Pumilio/Fem-3 binding factor (Puf) family Puf2, which is an RNA-binding protein (RBP), together with its interaction partner 7-helix-1, have been shown to be involved in translational repression of a number of gametocyte transcripts including Pfs25 and Pfs28 [13,16]. Moreover, the RBP Puf1 has been shown to play an important role in the differentiation and maintenance of female gametocytes [17].

To date, little is known of how the transcripts are regulated in male gametocytes. In previous studies, we carried out chemical loss of function studies using inhibitors targeting histone modification enzymes, and we showed significant deregulation of genes’ expression in immature, mature, and activated gametocytes following treatment with the inhibitors [18,19]. These studies indicated epigenetic gene regulation mechanisms during gametocyte development and potentially in male and female gametocytes. In one of the studies, we identified a CCCH-ZFP, which we named ZNF4 (PF3D7_1134600), that was highly deregulated following the treatment of the immature gametocytes with the histone deacetylase inhibitor Trichostatin A [18]. CCCH-type zinc finger proteins mainly act as RBPs with important roles in the RNA metabolism including RNA stability and transcriptional repression [20,21]. In some cases, CCCH-ZFPs may be trafficked between the nucleus and the cytoplasm [22] and bind both DNA and RNA [23], thereby functioning in both DNA and RNA regulation [24]. We now show that ZNF4 is a potential nucleic acid-binding protein which plays an important role in microgamete exflagellation through the regulation of male-specific genes during gametocyte development.

## 2. Materials and Methods

### 2.1. Antibodies

Antibodies used in this study included: rabbit anti-HA (Sigma Aldrich, Taufkirchen, Germany), rat anti-HA (Roche, Basel, Switzerland), mouse anti-GFP (Roche, Basel, Switzerland), mouse/rabbit anti-Pfs230 [25], rabbit/mouse anti-Pf39. Mouse anti-ZNF4 was generated for this study (see below). For indirect immunofluorescence assays (IFAs), the following dilutions of the antibodies were used: mouse/rabbit anti-Pfs230 (1:200), mouse anti-ZNF4 (1:20), mouse anti-GFP (1:200), rabbit anti-HA (1:50). For Western blot analysis, the following dilutions were used: rat/rabbit anti-HA (1:500), rabbit anti-Pf39 (1:10,000), mouse anti-GFP (1:1000).

### 2.2. Parasite Culture

The *P. falciparum* gametocyte-producing strain NF54 was used as background strain in this study. The parasites were cultivated in vitro in RPMI 1640/HEPES medium (Gibco, Thermo Scientific Waltham, MA, USA) supplemented with 10% heat-inactivated human serum and A^+^ erythrocytes at 5% hematocrit as described [26]. As supplement, 50 μg/mL hypoxanthine (Sigma Aldrich, Taufkirchen, Germany) and 10 μg/mL gentamicin (Gibco, Thermo Scientific Waltham, MA, USA) were added to the cell culture medium, and the cultures were grown in an atmosphere of 5% O_2_, 5% CO_2_, 90% N_2_ at a constant temperature of 37 °C. Cultures were synchronized by repeated sorbitol treatment as described [27].

Human erythrocyte concentrate and serum were purchased from the Department of Transfusion Medicine (University Hospital Aachen, NRW, Germany). The University Hospital Aachen Ethics commission approved all work with human blood, the donors remained anonymous, and serum samples were pooled.

### 2.3. Generation of Mouse Antisera

**Figure 1:**
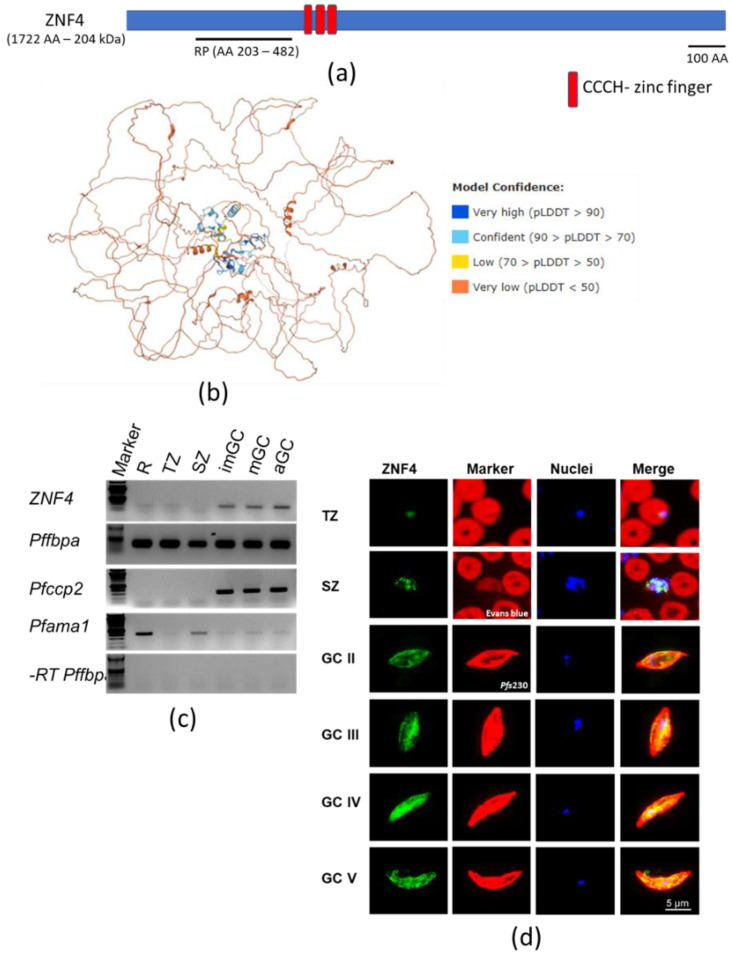
Domain architecture and protein expression of ZNF4: (**a**) Schematic of the ZNF4 domain structure. The 204 kDa protein contains three CCCH zinc finger domains indicated in red. The black line indicates the region used for the generation of the recombinant peptide. (**b**) Predicted 3D structure of ZNF4. The 3D structure of the protein was predicted using AlphaFold (https://alphafold.ebi.ac.uk/ accessed on 12 January 2022) [40,41]. Colours indicate the confidence level of prediction which ranged from blue (very high confidence) to orange (very low confidence). (**c**) Transcript expression of ZNF4 in the blood stages of *P. falciparum*. Complementary DNA was synthesized from rings (R), trophozoites (TZ), schizonts (SZ), immature (imGC), mature (mGC), and gametocytes at 30 min post-activation (aGC) and subjected to diagnostic PCR using ZNF4-specific primers. The expression levels of Pfama1 and Pfccp2 were used to verify asexual blood stage and gametocyte-specific expression. Samples lacking reverse transcriptase (-RT) were used as controls to check for any contamination with gDNA. Pffbpa was used as a loading control. (**d**) Localization of ZNF4 in different blood stages of *P. falciparum*. Anti-ZNF4 was used to immunolabel fixed samples of trophozoites (TZ), schizonts (SZ), and gametocytes (GC stage II to V) as well as of activated gametocytes (aGC) at 30 min post-activation (green). Asexual blood stages (trophozoites and schizonts) were visualized by labelling with Evans blue and gametocytes were visualized by using rabbit anti-Pfs230 (red); nuclei were highlighted by Hoechst nuclear stain 33342 (blue). Bar, 5 µm.

A recombinant protein, corresponding to a portion of ZNF4 (Figure 1a), was expressed as a maltose-binding tagged fusion protein using the pMAL™c5X-vector (New England Biolabs, Ipswich, MA, USA). The coding DNA sequence was amplified by PCR using gene-specific primers (for primer sequences, see Table S1). Recombinant protein was expressed in *E. coli* BL21 (DE3) RIL cells according to the manufacturer’s protocol (Invitrogen, Karlsruhe, Germany) and isolated and affinity-purified using amylose resin according to the manufacturer’s protocol (New England Biolabs, Ipswich, MA, USA). Polyclonal antisera were generated by immunization of 6-week-old female NMRI mice (Charles River Laboratories, Wilmington, NC, USA) subcutaneously with 100 µg recombinant protein emulsified in Freund’s incomplete adjuvant (Sigma Aldrich, Taufkirchen, Germany) followed by a boost after 4 weeks. At day 10 after the boost, mice were anesthetized by intraperitoneal injection of a mixture of ketamine and xylazine according to the manufacturer’s protocol (Sigma Aldrich, Taufkirchen, Germany), and immune sera were collected via heart puncture. The immune sera of three mice immunized were pooled; sera of three nonimmunized mice (NMS) were used as negative control. Experiments in mice were approved by the animal welfare committee of the District Council of Cologne, NRW, Germany (ref. no. 84-02.05.30.12.097 TVA).

### 2.4. Generation of Transgenic Parasite Lines

#### 2.4.1. Generation of ZNF4-KO Parasite Line

Disruption of *ZNF4* (PF3D7_1134600) was achieved by selection-linked integration as described [28]. Briefly, the plasmid pSLI-TGD-GFP was modified to contain a 601 bp homology block from the 5′ end of the *ZNF4* coding region (for primer sequence see Table S1). Parasites were transfected as described [18] and WR99210 (Jacobus Pharmaceuticals, Plainsboro Township, NJ, USA) was added at a final concentration of 4 nM, starting at 6 h after transfection, to select integrated parasites. WR99210-resistant parasites appeared after 21 days and were treated with a medium containing 400 μg/mL G418 (Sigma Aldrich, Taufkirchen, Germany); correct integration was confirmed by diagnostic PCR (for primer sequences, see Table S1). After successful integration was obtained, the lines were maintained through selection with 4 nM WR99210.

#### 2.4.2. Generation of ZNF4-HA-glmS Parasite Line

To generate a ZNF4-HA-glmS parasite line, we used selected linked integration insertion using a pSLI-HA-glmS vector (kindly provided by Dr. Ron Dzokowski, the Hebrew University of Jerusalem, Jerusalem, Isreal ), in which the plasmid was modified to contain a homology block from the 3′ end of the *ZNF4* gene, excluding the stop codon (for primer sequence, see Table S1). Parasites were transfected and WR99210 was added to a final concentration of 4 nM, starting at 6 h after transfection, to select integrated parasites. WR99210-resistant parasites appeared after 21 days and were treated with medium containing 400 μg/mL G418; correct integration was confirmed by diagnostic PCR (for primer sequences, see Table S1).

### 2.5. RNA-Based Methods

#### 2.5.1. RNA Isolation and RNA Sequencing

Total RNA was isolated from ring stage and Percoll-enriched mature (stage V) gametocytes from the ZNF4-KO and NF54 WT using the Trizol reagent (Invitrogen, Karlsruhe, Germany) according to the manufacturer’s protocol. Quality of RNA samples was assessed using an ND-1000 (NanoDrop Technologies, Thermo Scientific Waltham, MA, USA) and by agarose gel electrophoresis.

RNA sequencing was performed at the Genomic Facility of the University Clinic at the RWTH University Aachen, Germany. Briefly, the quality of the isolated total RNA samples from ring stage and mature gametocytes of the ZNF4-KO and the WT was evaluated by Tapestation 4200 (Agilent Technologies, Santa Clara, CA, USA). The quantities were measured by Quantus Fluorometer (Promega, Manheim, Germany). Libraries were generated with the TruSeq Stranded mRNA Library Preparation kit (Illumina) from high quality total RNA samples according to the manufacturer’s protocol. The generated libraries, which passed the quality control check on Tapestation 4200 (Agilent Technologies, Santa Clara, CA, USA), were sequenced on a NextSeq 500 (Illumina) with High output Kit v2.5 (150 cycles) for paired-end sequencing according to standard procedure provided by Illumina.

Data were analyzed with the NextGen pipeline, an in house-adapted pipeline embedded in the workflow management system of the QuickNGS-Environment [29]. In detail, the data were first demultiplexed according to corresponding indices. After quality assessment of the resulted fastq files with FastQC (v0.11.5; https://www.bioinformatics.babraham.ac.uk/projects/fastqc/ accessed 24 August 2019), STAR v2.5.2b [30] was applied to align the reads to the *P. falciparum* 3D7 EPr1 [31]) with default parameters. Aligned reads were quantified with Stringtie v1.3.6, as described [32]. Counts for transcripts were counted with featureCounts subread v1.5.1 [33], and differential expression analyses were finally conducted by comparing the transcript levels in the RNA samples of the ZNF4-KO and wildtype parasites for each stage using DESeq2 -R version 3.5.1 [34]. Raw data have been submitted to the NCBI Gene Expression Omnibus (GEO; http://www.ncbi.nlm.nih.gov/geo/ accessed on 30 April 2022) under accession number GSE196298.

#### 2.5.2. Semi-Quantitative RT-PCR

To determine the transcript expression of ZNF4, total RNA was isolated from rings, trophozoites, schizonts, and immature, mature, and 30 min post-activated gametocytes, as described above. One µg of each RNA sample was used for complementary DNA (cDNA) synthesis using the SuperScript IV First-Strand Synthesis System (Invitrogen, Karlsruhe, Germany), following the manufacturer’s instructions. The synthesized cDNA was first tested by diagnostic PCR for asexual blood stage contamination using specific primers, and controls without reverse transcriptase were also used to investigate potential genomic DNA (gDNA) contamination [19]. Transcript for ZNF4 (250 bp) was amplified using ZNF4 RT primers (for primer sequences, see Table S1). The following condition was used: initial denaturation at 94 °C for 2 min, followed by 25 cycles of denaturation at 94 °C for 30 s, of annealing at 45 °C for 30 s, and of elongation at 72 °C for 30 s, and a final extension at 72 °C for 2 min.

#### 2.5.3. Real-Time RT-PCR

To validate the RNA -Seq data, 1 µg of total RNA from mature gametocytes from the ZNF4-KO and WT parasite line was used for cDNA synthesis using the SuperScript IV First-Strand Synthesis System following the manufacturer’s instructions (Invitrogen, Karlsruhe, Germany). The synthesized cDNA was first verified by diagnostic PCR for the asexual blood stage and DNA contamination, using specific primers as described [18,19]. Primers for qRT-PCR corresponding to eight upregulated and three downregulated genes in the ZNF4-KO were designed using the Primer 3 software (http://frodo.wi.mit.edu/primer3/, accessed on 31 January 2020) and were tested in conventional PCR using DNA or cDNA to confirm primer specificity (for primer sequences, see Table S1). Real-time RT-PCR measurements were performed using the Step One Plus Real-Time Detection System (Thermo Scientific, Waltham, MA, USA). Reactions were performed in triplicate in a total volume of 20 μL using the maxima SyBR green qPCR master mix according to manufacturer’s instructions (Thermo Scientific, Waltham, MA, USA). Controls without template and without reverse transcriptase were included in all qRT-PCR experiments. The levels of transcript expression were calculated by the 2^−ΔCt^ method [35], using the endogenous control gene encoding the *P. falciparum* seryl tRNA-ligase (PF3D7_0717700) as reference [36,37].

### 2.6. Western Blotting

Asexual blood stage parasites of the WT or mutant parasite lines were obtained following tight synchronization of cultures with 5% sorbitol [27], while gametocytes were enriched by Percoll gradient purification [38]. Parasites were released from infected red blood cells (iRBCs) with 0.05% *w*/*v* saponin/PBS for 10 min at 4 °C, washed with PBS, and resuspended in lysis buffer (0.5% Triton X-100, 4% *w*/*v* SDS, 0.5 × PBS) supplemented with protease inhibitor cocktail (Roche, Basel, Switzerland). The lysates were then resuspended in 5 × SDS-PAGE loading buffer containing 25 mM dithiothreitol (DTT), heat-denatured for 10 min at 95 °C, and then separated via SDS-PAGE. After the proteins were separated, they were then transferred to the Hybond ECL nitrocellulose membrane (Amersham Biosciences, Buckinghamshire, UK), according to the manufacturer’s protocol. Membranes were blocked for nonspecific binding by incubation in Tris-buffered saline containing 5% skimmed milk and 1% BSA, followed by incubation with the respective primary antibody at 4 °C overnight. After washing, the membranes were incubated with an alkaline phosphatase-conjugated secondary antibody directed against the first antibody (Sigma Aldrich, Taufkirchen, Germany) for 1 h at RT and were developed in a solution of nitroblue tetrazolium chloride (NBT) and 5-bromo-4-chloro-3-indoxyl phosphate (BCIP; Sigma Aldrich, Taufkirchen, Germany) for 5–30 min.

### 2.7. Indirect Immunofluorescence Assay

Mixed cultures of the WT or mutant parasite lines were air-dried on glass slides and fixed for 10 min in a methanol bath at −80 °C. The RBCs were membrane permeabilized to allow access to the parasites and nonspecific binding sites were blocked by incubating the fixed cells in 0.01% saponin/0.5% BSA/PBS and 1% neutral serum each for 30 min at RT. Afterwards, the preparation was then incubated with the primary antibody diluted in 0.01% saponin/0.5% BSA/PBS for 2 h each at 37 °C. Binding of primary antibody was visualized by incubating the preparations with Alexa Fluor 488-conjugated secondary antibody directed against the primary antibody (Thermo Fisher Scientific, Waltham, MA, USA) diluted in 0.01% saponin/0.5% BSA/PBS for 1 h at 37 °C. The different parasite stages were detected through double-labelling with stage-specific marker primary antibodies or 0.001% *w*/*v* Evans blue (Sigma Aldrich, Taufkirchen, Germany), followed by incubation with Alexa Fluor 594-conjugated secondary antibodies (Thermo Fisher Scientific, Waltham, MA, USA) diluted in 0.01% saponin/0.5% BSA/PBS for 1 h at 37 °C. Nuclei were highlighted by treatment with Hoechst nuclear stain 33342 for 10 min at RT, and cells were mounted with anti-fading solution AF2 (Citifluor Ltd., Hatfield, PA, USA.) and sealed with nail polish. Digital images were taken using a Leica AF 6000 microscope and processed using Adobe Photoshop CS software.

### 2.8. Cell-Based Assays

#### 2.8.1. Asexual Blood Stage Replication Assay

To compare the asexual blood stage replication between the parental WT and the ZNF4-KO line, tightly synchronized ring stage cultures were set at an initial parasitaemia of 0.25%, and the development of the parasite was followed by Giemsa-stained thin blood smears that were prepared every 12 h over a time-period of 96 h at nine different time points (0, 12, 24, 36, 48, 60, 72, 84, 96 post-seeding). The parasitaemia of each time point was determined microscopically at 1000-fold magnification by counting the percentage of parasites in 1000 RBCs.

#### 2.8.2. Gametocyte Development Assay

To determine the effect of ZNF4-KO on gametocyte development, WT and ZNF4-KO parasite lines were tightly synchronized twice in two replication cycles and set to a parasitaemia of 5% ring stage parasites. Gametocytogenesis was then induced by the addition of lysed RBCs (0.5 mL of 50% haematocrit lysed RBC in 15 mL of culture medium) followed by washing of the cell the next day. The cultures were then maintained in cell culture medium supplemented with 50 mM N-acetylglucosamine (GlcNAc) to kill the asexual blood stages for 5 days [39] and then maintained with normal cell culture medium until day 10 post-induction. Samples were taken in triplicate every 24 h starting from day 5 post-induction for Giemsa smear preparation. Gametocytaemia was determined per 1000 RBCs, and the gametocyte stages II–V at the different time points from 50 gametocytes were counted in triplicate. For each assay, two experiments were performed, each in triplicate.

#### 2.8.3. Exflagellation Assay

To determine the effect of *ZNF4* disruption on the ability of male gametocytes to exflagellate, gametocytaemia of matured gametocytes of the WT and the ZNF4-KO parasite lines were determined. Next, 100 µL of each gametocyte culture was activated in vitro with 100 µM xanthurenic acid (XA) for 15 min at RT. After activation, the numbers of exflagellation centres were counted at 400-fold magnification in 30 optical fields using a Leica DMLS microscope and the number of exflagellation centres were adjusted with the gametocytaemia. Exflagellation was calculated as a percentage of the number of exflagellation centres in the ZNF4-KO in relation to the number of exflagellation centres in the WT control (WT set to 100%).

#### 2.8.4. Membrane-Feeding Assay

The effect of *ZNF4* depletion on malaria transmission was performed using membrane-feeding assays at the TropIQ Health Science, Nijmegen, The Netherlands, through the support of the Infrastructure for the control of vector borne diseases (infravec2). Briefly, gametocyte cultures of the WT and the ZNF4-KO lines were set up and, on day 16, when the gametocytes were fully matured, 10^6^/mL gametocytes were fed to female *Anopheles stephensi* mosquitoes using standard membrane-feeding assays. After 7 days, the midguts were dissected, and the oocysts were counted following staining with mercurochrome.

### 2.9. Statistical and Online Analysis

Statistical analysis of significant differences in exflagellation, occyst load, and percentage of infected mosquitoes between WT and ZNF4-KO was performed using the student´s t-test in the Graph Pad Prism software. The ZNF4 domain structure and 3D structure were predicted using UniProt (https://www.uniprot.org/uniprot/Q8II18, accessed on 12 January 2022) and AlphaFold [40,41], respectively. Gene ontology (GO) enrichment analyses were determined using PlasmoDB (plasmodb.org/plasmo/app, accessed on 1 January 2022). For GO analysis, the default settings were used with *p* < 0.05. To compare transcript expression of the top 30 downregulated genes, a heat map was constructed using TB tools [42].

To determine the enriched motifs in the deregulated genes, the comprehensive motif analysis tool XSTREME was used (meme-suite.org, [43]). The top 2 enriched motifs between 7 and 15 nucleotides in the deregulated genes, as compared to controls (48 genes with fold change 1.00), were considered. Transcripts of deregulated genes as well as control were downloaded from the PlasmoDB (plasmodb.org/plasmo/app, accessed on 13 January 2022).

## 3. Results

### 3.1. ZNF4 Is a CCCH-ZFP Expressed Mainly in Gametocytes of P. falciparum

Analysis of the ZNF4 protein features using UniProt shows a 204 kDa protein with three CCCH zinc finger domains between amino acid 513 to 540, 548 to 574, and 582 to 610 (Figure 1a). In addition, the 3D structure of the protein, as predicted using AlphaFold [40,41], shows the arrangement of alpha helices and the beta strands in the CCCH domains to coordinate the zinc binding (Figure 1b). To determine the transcript expression of ZNF4, a semi-quantitative RT-PCR was performed using asexual blood stages (rings, trophozoites and schizonts) as well as gametocyte stages (immature, mature, and 30 min post-activated gametocytes). The transcript levels of Pfama1 (apical membrane antigen 1) and Pfccp2 (LCCL domain-containing protein) were determined to control for purity in asexual blood stage and gametocyte samples, respectively. Samples without reverse transcriptase (-RT) were used as controls to verify the absence of gDNA. Pffbpa (fructose-bisphosphate aldolase) was used as housekeeping loading control. High transcript expression of ZNF4 was observed in the gametocyte stages as compared to the asexual blood stages (Figure 1c). To determine the ZNF4 protein expression, a recombinant peptide (RP) corresponding to a portion of ZNF4 (Figure 1a) was expressed in *E. coli* and used to generate mouse polyclonal antisera against ZNF4. Immunofluorescence assays (IFAs) show mainly a cytoplasmic expression of ZNF4 in both asexual blood stages and gametocytes, with the highest expression occurring in mature gametocytes (Figure 1d). No differences in sex-specific expression between male and female gametocytes were observed (data not shown). To confirm ZNF4 expression, we used a pSLI-ZNF4-HA-glmS parasite line in which the 3′ end of the endogenous gene was fused with the sequence of a 3× HA-tag and a glmS ribozyme (Figure S1a). Successful integration in the parasite line was obtained (Figure S1b), and we were able to detect the HA-tagged ZNF4 in mature gametocyte lysates at a molecular weight of roughly 250 kDa (Figure S1c). IFAs using anti-HA confirmed the expression of the protein in the asexual blood stages and in gametocytes, with the highest expression occurring in mature gametocytes (Figure S1d).

### 3.2. Targeted Gene Disruption of ZNF4 Does Not Impact Asexual Blood Stage Replication and Gametocyte Development

**Figure 2:**
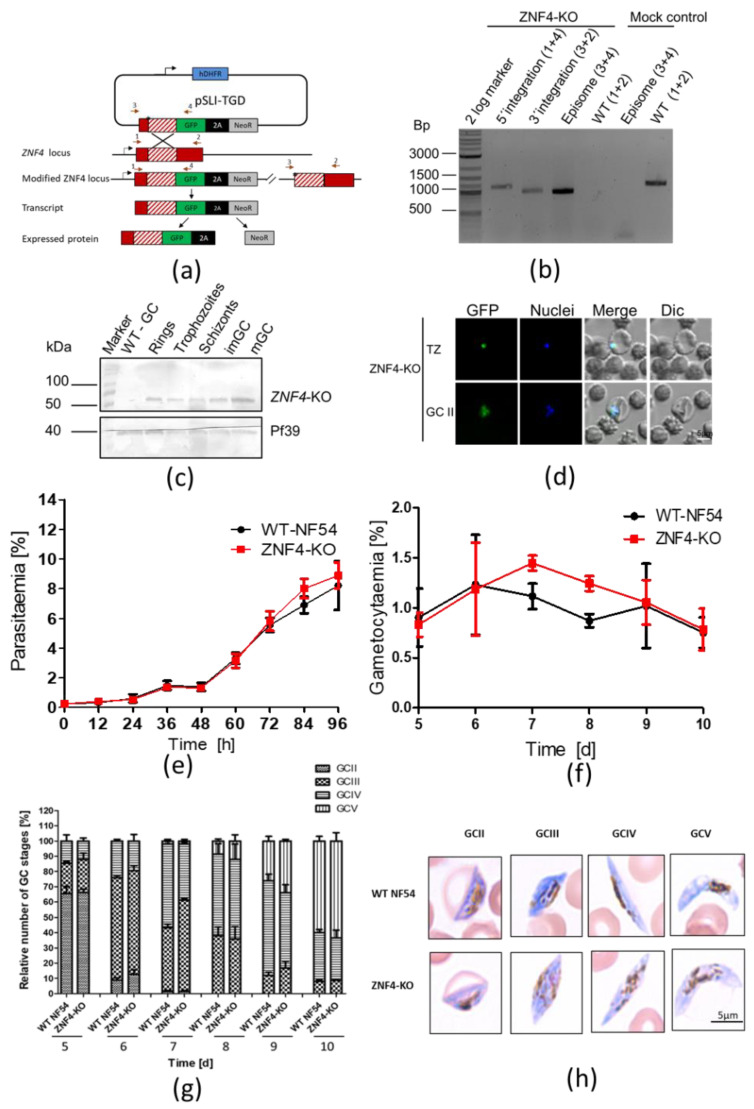
Targeted gene disruption of ZNF4 and its effect on asexual blood stage replication and gametocytogenesis: (**a**) Schematic depicting the generation of ZNF4-KO via single crossover recombination-based gene disruption using selective linked integration targeted gene disruption (SLI-TGD). The vector pSLI-TGD was modified to contain a 601 bp sequence block (white box with red stripes) from near the 5′ end of the ZNF4 coding region (red box). The coding region was maintained in frame with a GFP protein coding region (green box), a 2A “skip” peptide (black box), and the Neo-R gene (grey) that provides resistance to the antibiotic G418. Arrows indicate the position of primers 1–4, used to detect integration of the pSLI-TGD vector. Asterisks indicate a stop codon. GFP, green fluorescent protein; hDHFR; human dehydrofolate reductase for resistance to WR99210; NeoR, neomycin-resistance; 2A, skip peptide. (**b**) Confirmation of vector integration for the ZNF4-KO parasites by diagnostic PCR using gDNA obtained from ZNF4-KO and WT-NF54. 5′-integration was detected using primers 1 and 4 (1164 bp) and 3′-integration using primers 3 and 2 (955 bp). Primers 3 and 4 were used to detect the presence of episome (965 bp), and primers 1 and 2 were used for WT control (1194 bp). (**c**) Confirmation of truncated ZNF4 tagged with GFP. Parasite lysates obtained from different stages of the ZNF4-KO parasite line were subjected to Western blotting using polyclonal mouse anti-GFP (estimated size 53 kDa). Lysate from WT mature gametocyte was used as negative control. Immunoblotting with mouse anti-Pf39 antisera (39 kDa) served as a loading control. (**d**) Verification of GFP expression in the ZNF4-KO parasites by live imaging. Live images of trophozoites (TZ) and gametocyte stage II (GCII) of the ZNF4-KO line detected GFP (green) in the parasite. Dic, differential interference contrast. Nuclei were counterstained with Hoechst 33342 (blue). Bar, 5 µm. (**e**) Asexual blood stage replication of the ZNF4-KO. Synchronized ring stage cultures of WT and ZNF4-KO with an initial parasitaemia of 0.25% were maintained in cell culture medium, and the parasitaemia was followed over a time-period of 0 to 96 h via Giemsa stained smears. The data are a representation of one of two experiments performed in triplicate (mean ± SD). For the second experiment, see Figure S2a. (**f**) Disruption of ZNF4 shows no effect in gametocytaemia. Following two rounds of synchronization, a culture of 5% of the ring stage parasites of the WT and ZNF4-KO was induced for gametocytogenesis and, the next day, the parasites were washed and grown with medium supplemented with 50 mM GlcNac to kill asexual blood stages for 5 days, then with normal medium until day 10 post-induction. The gametocytaemia was monitored by Giemsa-stained blood smears from day 5 post-induction. The result is a representative of one of two experiments (see Figure S2b for second experiment). (**g**) Gametocyte maturation in the ZNF4-KO. The development of gametocyte was compared between the WT and the ZNF4-KO by counting the gametocyte stages of 50 gametocytes at each time point in triplicate (See Figure S2c for second experiment). (**h**) Gametocyte morphology in the ZNF4-KO line. Giemsa-stained pictures of gametocyte stages of the ZNF4-KO and WT.

To determine the function of ZNF4, we utilized the selected linked integration-mediated targeted gene disruption method [28] to generate a disrupted ZNF4 parasite line, named ZNF4-KO (Figure 2a). These parasites express only a truncated N-terminal GFP tagged fragment which lacks the three CCCH zinc finger domains. Successful disruption was confirmed by diagnostic PCR, which indicated 5′ and 3′ integration and a lack of wildtype in the ZNF4-KO parasite line (Figure 2b). To further confirm the gene disruption, the remaining part of the ZNF4-KO truncated protein which is fused to GFP was detected by Western blotting using different parasite stages (rings, trophozoites, schizonts, and immature and mature gametocytes) as well as by live imaging (Figure 2c,d). The Western blotting with the ZNF4-KO parasite line further confirmed the expression of the truncated protein at the expected molecular weight of 53 kDa in different *P. falciparum* stages (Figure 2c).

After successful gene disruption, we first examined the asexual blood stage replication in the ZNF4-KO line. To this end, the development of highly synchronized ring stages of the ZNF4-KO and WT parasite lines was monitored over a period of 96 h by Giemsa-stained blood smears, which were taken every 12 h. The results showed no significant difference in intraerythrocytic replication between the ZNF4-KO and the WT (Figure 2e). Then, ring stage parasites, after the second replication cycle, were induced for gametocytogenesis, and asexual blood stages were eliminated. Gametocyte development and gametocytaemia were followed from day 5 post-activation until day 10. No significant effect in gametocytaemia and gametocyte development was observed (Figure 2f,g). In addition, the morphology of ZNF4-KO gametocytes was not affected (Figure 2h).

### 3.3. Disruption of ZNF4 Impacts gametocyte Exflagellation and Reduces Transmission in the Mosquito

**Figure 3:**
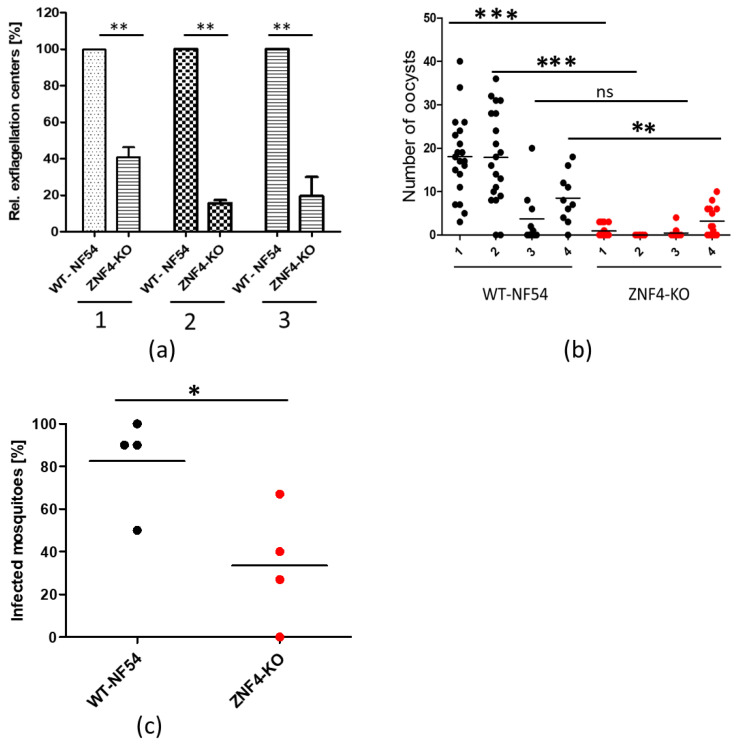
ZNF4-KO impairs male gametocyte exflagellation and parasite transmission in the mosquito: (**a**) Disruption of ZNF4 impairs male gametocyte exflagellation. Mature WT and ZNF4-KO gametocytes were activated in vitro and the number of exflagellation centres was counted in 30 fields in triplicate using the light microscope. Three independent experiments were performed, indicated in numbers 1 to 3. **, *p* < 0.01, Student’s *t* test. (**b**) Mosquito infectivity of ZNF4-KO. Enriched mature gametocytes of WT or the ZNF4-KO were fed to An. stephensi mosquitoes via standard membrane feeding assays. The number of oocysts per midgut were counted at day 7 post-infection in four independent experiments; ***, *p* < 0.001, **, *p* < 0.01; ns, not significant, Student’s *t* test. (**c**) Percentage of infected mosquitoes in WT versus ZNF4-KO in each standard membrane-feeding experiment; * *p* < 0.05, Student’s *t* test.

To determine whether male gametocytes of the ZNF4-KO parasite line are able to produce motile microgametes, an in vitro exflagellation assay was performed. To this end, mature gametocytes of the ZNF4-KO and WT were activated with xanthurenic acid (XA) for 15 min at RT. After activation, the number of exflagellation centres were counted microscopically. The results show that the ZNF4-KO produced significantly lower numbers of exflagellation centres as compared to WT (Figure 3a), indicating an impairment in male gametocyte exflagellation. To determine if this impairment can affect malaria transmission in the mosquitoes, the matured gametocytes were fed to Anopheles stephensi mosquito in membrane-feeding assays and the number of oocysts were counted. Although the ZNF4-KO line still produced oocysts, their numbers were relatively very low as compared to the WT (Figure 3b). Moreover, there was a significant difference in the prevalence of mosquito infection between WT and ZNF4-KO when the standard membrane-feeding experiments were compared (Figure 3c).

### 3.4. ZNF4 Disruption Results in DownRegulation of Male Gametocyte-Enriched Transcripts

**Figure 4:**
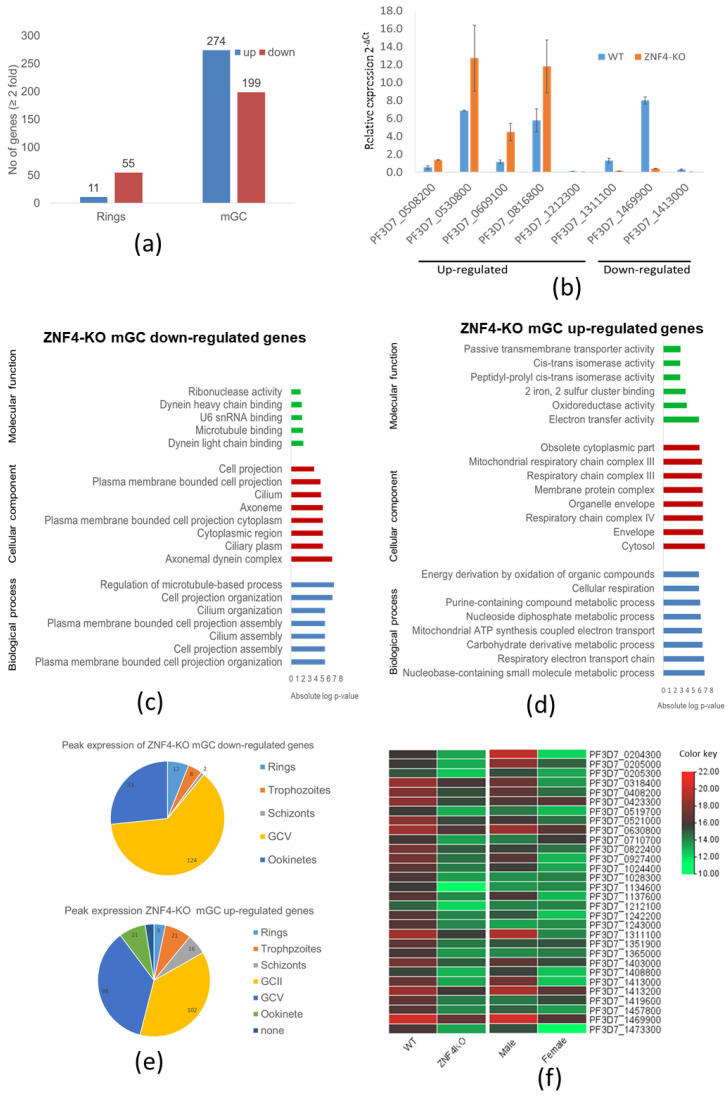
Deregulation of gene expression following disruption of ZNF4: (**a**) Comparative transcriptomics of deregulated genes in the ZNF4-KO parasite line in rings and mature gametocytes. Total RNA was extracted from ring stage parasites as well as the mature gametocytes of the ZNF4-KO and the WT and were subjected to comparative transcriptomics by RNA sequencing. Deregulated genes with relative gene expression greater than 2-fold were considered significant. (**b**) Validation of RNA-Seq data by qRT. Transcript analysis for 5 upregulated genes and 3 downregulated in mature gametocyte, as identified by RNA-Seq, were validated by real-time RT-PCR. Transcript expression levels were calculated by the 2^−ΔCt^ method; the threshold cycle number (Ct) was normalized with the Ct of the gene encoding seryl tRNA-ligase (PF3D7_0717700) as reference. Genes were considered upregulated when the changes between ZNF4-KO and WT sample were greater than 2-fold. (**c**,**d**) Summary of Gene Ontology functional analysis of mature gametocyte differentially expressed genes following ZNF4 disruption. GO enrichment analysis of deregulated genes was determined using PlasmoDB (https://plasmodb.org/plasmo/app, accessed on 1 January 2022). The most significantly (*p* < 0.05) enriched GO terms in the biological process, the cellular component, and the molecular function are presented. For the complete list, see Table S2. All adjusted statistically significant values of the terms were the absolute log_10_ values. GO, gene ontology. (**e**) Pie chart showing peak expression stage of deregulated genes. The peak expression of the downregulated as well as the upregulated genes were determined using the 7 stage RNA-Seq data [44]. (**f**) Heat map of top 30 downregulated genes and their sex-specific expression. Heat map representing patterns of the top 30 downregulated genes and their sex-specific expression pattern [45]. Heat map was constructed using TB tools [42].

To compare the transcriptome of the ZNF4-KO and the WT, we carried out a comparative RNA-Seq using ring stage parasites and mature gametocytes of the ZNF4-KO and the WT. We used a cut off greater than a 2-fold change in expression. The results show that 66 genes were deregulated in the ring stage following ZNF4 disruption (55 downregulated and 11 upregulated) as opposed to mature gametocytes, in which a total of 473 genes (274 upregulated and 199 downregulated) were detected (Figure 4a, Table S2). For further analysis, we then focused on the deregulated genes in mature gametocytes, since the knockout phenotype suggests that male gametocyte exflagellation was affected. To validate the RNA-Seq data, we carried out a quantitative real-time experiment (qRT) to compare the transcript expression of eight genes (five upregulated and three downregulated) using RNA from the mature gametocytes of the ZNF4-KO and the WT. The results confirmed the RNA-Seq data, as four of the five upregulated genes in the RNA-Seq data were upregulated and all downregulated genes were downregulated in the qRT (Figure 4b).

We next performed a gene ontology (GO) enrichment analysis (Table S2). Downregulated genes could mainly be assigned to biological processes such as the regulation of microtubule-based processes, cell projection organization, and cilium organization (Figure 4c). Regarding cellular components, axonemal dynein complex assembly was highly represented, and, for molecular function, dynein light chain binding and microtubule binding was also highly represented (Figure 4c). Upregulated genes were mainly assigned to nucleoside containing small molecule metabolic processes and the respiratory electron transfer chain as biological processes. In addition, the respiratory chain complex, the mitochondrial respiratory chain complex (cellular component), as well as electron transfer activity and oxidoreductase activity (molecular function), were the mostly represented (Figure 4d).

We also determined at which stage of the parasite life cycle does the deregulated genes show peak expression according to the seven stage RNA-Seq data [44]. We observed that most of the downregulated genes show peak expression in stage V gametocytes, followed by the ookinete stage (Figure 4e). On the other hand, the upregulated genes showed peak expression in stage II and stage V gametocytes (Figure 4e).

Since the disruption of ZNF4 mainly affected male gametocyte exflagellation, we analyzed if the effect was due to the downregulation of transcripts enriched in male gametocytes. For this reason, we compared the sex-specific transcript expression of the top 30 downregulated genes using the sex specificity data from Lasonder and colleagues [45]. The heat map shows that, indeed, most of the downregulated genes have high transcript levels in male gametocytes with genes such as PF3D7_1469900 (male gametocytes enriched transcribe, MGET) and PF3D7_1311100 (meiosis-specific nuclear structural protein 1, putative) (Figure 4f).

### 3.5. ZNF4-KO Upregulated and Downregulated Genes Show Different Predicted Enriched RNA-Binding Motifs

To determine the predicted RNA-binding motif in the deregulated mature gametocyte genes, the comprehensive motif analysis tool XSTREME (Meme-suite.org [43]) was used to check which motifs were enriched in the upregulated and downregulated genes as compared to the control transcripts which were not affected. We used downloaded transcript sequences from the PlasmoDB website (plasmodb.org/plasmo/app, accessed 21 January 2022) containing the 5′ and 3′UTR and searched for the motif of 7 to 15 nucleotides. The upregulated genes motifs identified were U-rich, with the top two hits being “UUUUUUUUUUUUAU”, with this signature found in 199 of the 274 upregulated transcripts (e-value:1.8 × 10^−15^; Table 1), and “AUUUUUAUUUU”, with this signature found in 207 of the 274 genes (e-value:1.5 × 10^−10^; Table 1). On the other hand, motifs of the downregulated genes were mainly A-rich, with top hits being “AAAAUAUAAAAAAA”, with this signature in 138 of the 199 downregulated genes (e-value:3.3 × 10^−12^; Table 1), and “AAAAAAAAGAAAA”, with signature in 165 out of the 199 downregulated genes (e-value:5.8 × 10^−11^; Table 1). Interestingly, these motifs were highly similar to known RBP motifs (Table 1). This indicates that ZNF4 is probably binding to different motifs in the upregulated and downregulated genes.

## 4. Discussion

The highly complex life cycle of the human malaria parasite *P. falciparum* requires a well-coordinated gene regulation to allow for gametocyte commitment, development, and human-to-mosquito transmission of the parasite. Translational repression in particular has been shown to be the main player in preparing female gametocytes for parasite transmission, with RNA-binding translational repressors such as DOZI, CITH, or Puf2 playing crucial roles. While the mechanism by which female gametocytes genes are regulated has been studied in detail, the mechanism of the regulation of male-specific genes remains largely unknown. In the present study, we show that the PfCCCH ZFP, named ZNF4, is a potential RBP that is important for male gametogenesis and hence malaria transmission through the regulation of male-enriched gametocyte genes. Noteworthy, ZNF4 is expressed mainly in the cytoplasm of both male and female gametocytes in accordance with previous sex specificity data [45], indicating that ZNF4 expression is not dependent of the gametocyte sex of the parasite.

Targeted gene disruption of *ZNF4* showed normal progression through the intraerythrocytic replication cycle, indicating that the gene is dispensable for asexual blood stage replication. This is not surprising, as a recent genome-wide transposon mutagenesis screen in *P. falciparum* also indicates that the gene is not essential for parasite viability [47]. Moreover, the ZNF4-KO parasite line did not show any significant effect in gametocyte formation and development, with the gametocytes showing normal morphology. However, although gametocyte development was not affected, *ZNF4* disruption greatly impaired malaria transmission in the mosquito through the inhibition of exflagellation and, in consequence, oocyst formation. A recent study also associated the involvement of a *P. berghei* RNA-binding protein, UIS12, to gametocyte exflagellation and malaria transmission [48]. The fact that *ZNF4* disruption only led to a partial and not complete alteration in gametocyte exflagellation, which resulted in a significant reduction in oocyst load, indicates that some of the remaining exflagellating ZNF4-KO male gametocytes may produce microgametes which are defective in female gamete fertilization.

To determine the possible cause of exflagellation inhibition in the ZNF4-KO, we carried out comparative transcriptomic analysis in mature gametocytes as well as in the ring stages. Only a few genes (66) were >2-fold deregulated in the ring stage following *ZNF4* disruption, which is in accordance with the lack of any phenotype during asexual blood stage replication, indicating that ZNF4 has no special role in ring stage parasites. Moreover, 473 genes were deregulated in ZNF4-KO mature gametocytes (274 genes upregulated and 199 downregulated), pointing to the effect in male gametocyte exflagellation.

Further analysis demonstrated that the majority of the downregulated genes exhibit peak expression in mature gametocytes, where they are implicated in essential cellular and biological processes linked to male gametocyte exflagellation such as cell projection assembly, cilium assembly, and the axonemal dynein complex. The upregulated genes mainly exhibit peak expression in stage II gametocytes, with represented cellular and biological processes being associated to the respiratory electron chain and mitochondrial ATP synthesis. Interestingly, a previous study that integrated transcriptomics and proteomic, associated male gametocytes to be enriched in proteins linked to the formation of flagellated gametes, axoneme formation, DNA replication, and chromatin organization, while female gametocytes were enriched in proteins associated with protein, lipid, and energy metabolism [45]. The downregulation of genes mainly associated to cell projection assembly, cilium assembly, and axonemal dynein complex formation therefore justifies the defect in male gametocyte exflagellation, and studies in other organisms have associated the processes to proper flagella formation, movement, and fertility [49,50]. Regarding the upregulated genes following ZNF4-KO, it is likely that, since energy metabolism is not very necessary for male gametocytes, these genes are being repressed in male gametocytes by ZNF4, and they become upregulated following ZNF4 disruption. In accordance with our findings, the top 30 downregulated genes showed high expression in male gametocytes. Although the function of most of the highly expressed male genes are unknown, some prominent genes encode for PF3D7_1469900 (PfMGET), an abundant protein transcribed specifically in male gametocytes, which has been used for male gametocyte quantification [51,52] and for PF3D7_1311100 (meiosis-specific nuclear structural protein 1, putative) with human homolog MSN1, which has been linked to male fertility [53,54].

An interesting finding in this study is the fact that upregulated genes and downregulated genes possess different binding motifs, suggesting that they may be regulated differently. The motifs for the upregulated genes have been reported to bind RBPs such as SXL and HuR. SXL, also known as sex lethal, is a master regulator of sex determination in *Drosophilia melanogaster* by regulating the choice between male and female development pathways [55,56]. For the downregulated gene motifs, they have been reported as targets for RBPs such as Nab2p and PABC4. Nabp2 in *Saccharomyces cerevisiae* is a nuclear protein required to protect early mRNA, and has also been reported to be involved in RNA exportation from the nucleus to the cytoplasm [57,58]. PABC4 (cytoplasmic poly(A) binding protein C4) plays a critical role in erythroid differentiation through mRNA regulation [59].

The exact mechanism by which ZNF4 regulates gene expression warrants further investigation. One potential mechanism could be by translational repression, as has been shown for female gametocytes, where mRNA transcripts important for zygote/ookinete development, such as P25 and P28, are stored in a messenger ribonucleoprotein complex composed of RBPs such as DOZI and CITH in *P. berghei* [14,15] or Puf2 and 7-Helix-1 in *P. falciparum* [13,60]. Noteworthy, a recent study in *P. berghei* has identified a CCCH domain containing ZFP—Pb103 associated to zygote/ookinete development, probably by translational repression [61]. It is likely that there exists a translational repression mechanism regulating male gametocyte genes in which ZNF4 is part of the complex.

Another probable mechanism by which ZNF4 controls transcripts is by the regulation of mRNA stability, as has been reported for many CCCH-ZFPs, such as the tristretraprolins (TTPs), which are the most studied CCCH-ZFPs. TTPs have been shown to bind AU-rich elements in mRNAs, resulting in the removal of the poly-A tail from the mRNA, thereby marking them for decay [62]. It is possible that the deficiency of ZNF4 leads to the accumulation of transcripts due to the absence of mRNA stabilization, as observed with the upregulated genes.

## 5. Conclusions

We have identified a novel ZFP, ZNF4, and have shown that ZNF4 plays an essential role in gametocyte exflagellation and, consequently, parasite transmission in the mosquito. The defect in gametocyte exflagellation observed in the ZNF4-KO was justified by RNA-Seq, demonstrating the downregulation of male gametocyte-enriched genes mainly associated with axonemal dynein complex formation, cilium assembly, and cell projection organization. This combined data indicates that ZNF4 is important for gametocyte exflagellation through the regulation of male transcripts. However, further studies will be required to address the mechanism by which ZNF4 regulates these genes and if more critical regulatory proteins are involved.

## Figures and Tables

**Table 1:** Predicted binding motif for ZNF4-KO in the upregulated and downregulated genes.

ZNF4-KO	Predicted Motif	No of Positive Genes (%)	E-Value	Similar Known Motif [46]
Up-regulated genes	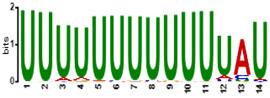	199 (72.6%)	1.8 × 10^−15^	SXL (RNCMPT00119) Pp_0228 (RNCMPT00228) Tv_0236 (RNCMPT00236)
	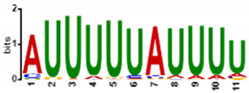	207 (75.5%)	1.6 × 10^−12^	HuR (RNCMPT00032) HNRNPC (RNCMPT00025) HNRNPCL1 (RNCMPT00167
Down-regulated genes	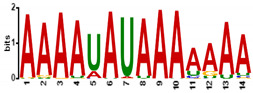	138 (69.3%)	3.3 × 10^−12^	KHDRBS1 (RNCMPT00169) Lm_0255 (RNCMPT00255) PABPC4 (RNCMPT00043
	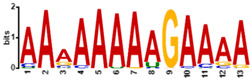	165 (82.9%)	5.8 × 10^−11^	Nab2p (RNCMPT00042) Hnrnpr (RNCMPT00289) PABPC4 (RNCMPT00043)

## Data Availability

RNA-Seq raw data have been submitted to the NCBI Gene Expression Omnibus (GEO; http://www.ncbi.nlm.nih.gov/geo/ accessed on 30 April 2022) under accession number GSE196298.

## Supplementary Materials

The following supporting information can be downloaded at: www.mdpi.com/article/10.3390/cells11101666/s1. Figure S1: Validation of ZNF4 protein expression using the pSLI-ZNF4-HA-glmS parasite line; Figure S2: Phenotypic characterization for ZNF4-KO; Table S1: List of primers used in the study; Table S2: List of ZNF4-KO deregulated genes in gametocytes and rings and complete GO-Analysis.
