# Supplementary material for: The Plasmodium falciparum CCCH Zinc Finger Protein ZNF4 Plays an Important Role in Gametocyte Exflagellation through the Regulation of Male Enriched Transcripts
Source: Cells. 2022 May 17;11(10):1666. doi: 10.3390/cells11101666 (PMC9139750; doi:10.3390/cells11101666)
Supplement: Supplementary file 1 [file cells-11-01666-s001.zip › Supplimentary Figures.pdf]

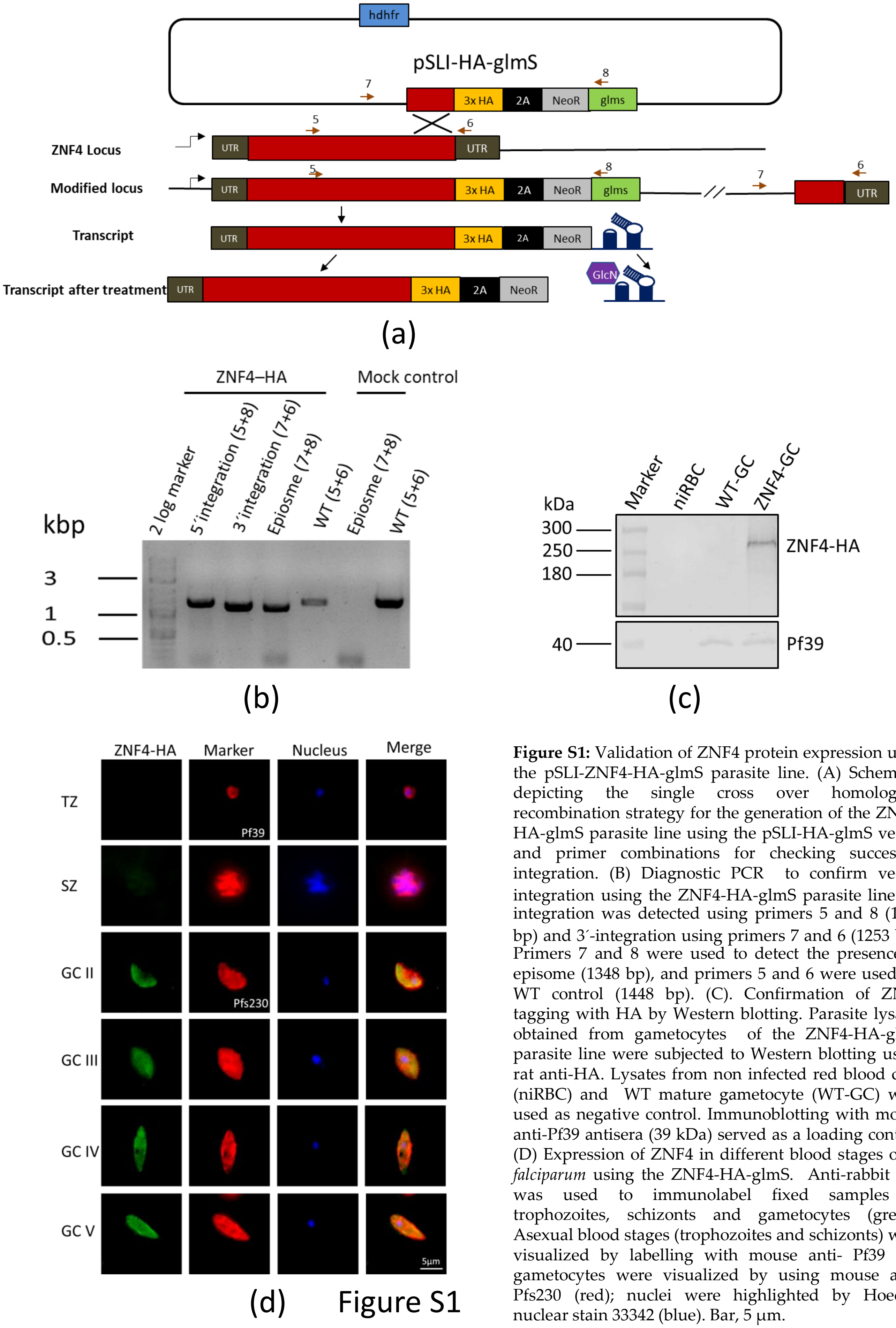

Figure S1: Validation of ZNF4 protein expression using the pSLI-ZNF4-HA-glmS parasite line. (A) Schematic depicting the single cross over homologous recombination strategy for the generation of the ZNF4-HA-glmS parasite line using the pSLI-HA-glmS vector and primer combinations for checking successful integration. (B) Diagnostic PCR to confirm vector integration using the ZNF4-HA-glmS parasite line. 5´-integration was detected using primers 5 and 8 (1543 bp) and 3´-integration using primers 7 and 6 (1253 bp). Primers 7 and 8 were used to detect the presence of episome (1348 bp), and primers 5 and 6 were used for WT control (1448 bp). (C). Confirmation of ZNF4 tagging with HA by Western blotting. Parasite lysates obtained from gametocytes of the ZNF4-HA-glmS parasite line were subjected to Western blotting using rat anti-HA. Lysates from non infected red blood cells (niRBC) and WT mature gametocyte (WT-GC) were used as negative control. Immunoblotting with mouse anti-Pf39 antisera (39 kDa) served as a loading control. (D) Expression of ZNF4 in different blood stages of *P. falciparum* using the ZNF4-HA-glmS. Anti-rabbit HA was used to immunolabel fixed samples of trophozoites, schizonts and gametocytes (green). Asexual blood stages (trophozoites and schizonts) were visualized by labelling with mouse anti- Pf39 and gametocytes were visualized by using mouse anti-Pfs230 (red); nuclei were highlighted by Hoechst nuclear stain 33342 (blue). Bar, 5 µm.

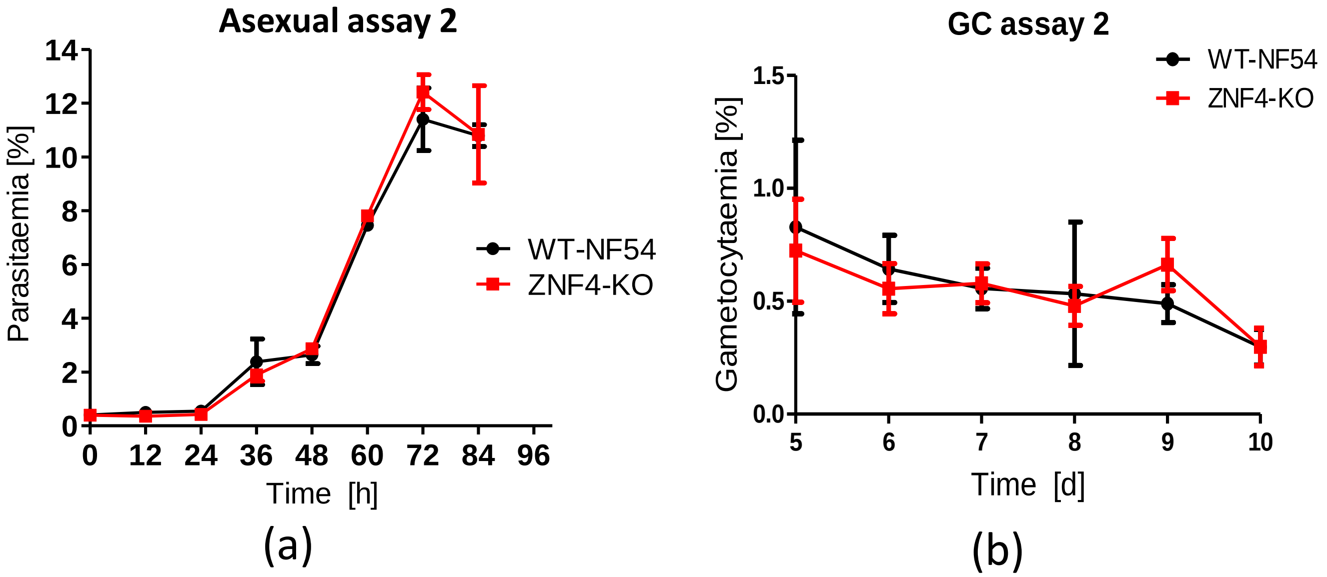

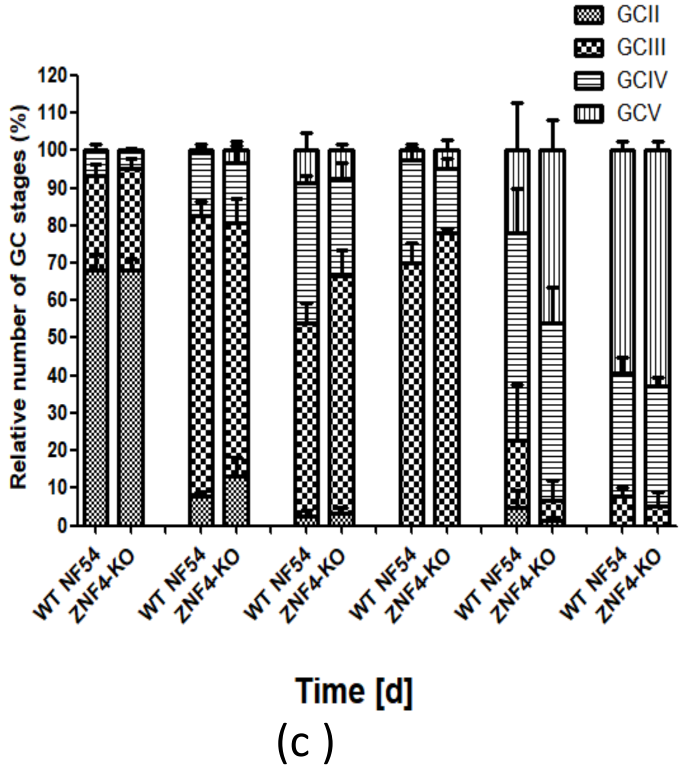

Figure S2: Phenotypic characterization for ZNF4-KO. Second experiment for the Asexual development (A), Gametocytaemia (B) and gametocyte development (C) of the ZNF4-KO as compared to WT.

Figure S2
